# Motivation as the driving force of functioning in psychotic disorders

**DOI:** 10.1192/j.eurpsy.2025.2057

**Published:** 2025-08-26

**Authors:** E. Rosado, A. M. Sánchez-Torres, G. Gil-Berrozpe, J. Chato, X. Ansorena, A. Zarzuela, J. I. Arrarás, M. J. Cuesta

**Affiliations:** 1 Instituto de Investigación Sanitaria de Navarra; 2 Servicio de Psiquiatría, Hospital Universitario de Navarra Basque Center on Cognition, Brain and Language; 3 Departamento de Ciencias de la Salud, Universidad Pública de Navarra; 4 Clínica de Rehabilitación de Salud Mental, Hospital Universitario de Navarra, PAMPLONA, Spain

## Abstract

**Introduction:**

In the last decades, research has focused on investigating cognition and psychopathological symptoms as variables contributing to functional outcomes. However, in recent years the study of motivation has attracted interest as a research target related to functional outcome (Miley *et al* Psychol Med. 2023;53(5):2041-2049).

**Objectives:**

We aimed to study the relationship of clinical symptoms, motivation, socio-affective capacity and cognition with functioning in social and occupational areas in patients with a psychotic disorder.

**Methods:**

A sample of 97 patients with a DSM-5 psychotic disorder diagnosis was included. Assessments included the Specific Levels of Functioning Scale (SLOF; Schneider *et al.* Soc Work Res Abstr 1983;19(3):9-21) to assess functioning; the Comprehensive Assessment of Symptoms and History (CASH; Andreasen *et al*. Arch Gen Psychiatry 1992; 49(8):615-23) for clinical symptoms; and the Cognitive Assessment Interview (CAI-Sp; Ventura *et al.* Schizophr Res 2010; 121(1-3): 24-31) and a comprehensive neuropsychological battery to assess cognition. Motivation and socio-affective capacity were assessed by means of the Quality of Life Scale (QLS; Heinrichs *et al*. Schizophr Bull. 1984; 10(3):388-98). Both domains were derived from items of the Intrapsychic Foundations subscale of the QLS. Motivation was derived from items 13, (sense of purpose), 14 (degree of motivation) and 15 (curiosity). Socio-affective capacity comprised items 20 (capacity for empathy) and 21 (capacity for engagement and emotional with the interviewer).

Spearman correlations were calculated. Variables which correlated significantly (p<0.05) with SLOF scores were included in the regression analyses.

**Results:**

All the clinical, cognitive and related with motivation and socio-affective capacity variables included in the analyses were significantly correlated with SLOF scores (Table 1), except for positive symptoms with SLOF activities and work. However, in the hierarchical analyses most of the variables were not significant. Specifically, regarding SLOF social scores, positive symptoms and motivation explained 51.5% of the variance. Motivation also explained 40.1% and 68% of the variance of the scores of SLOF activities and work, respectively (Table 2).

**Image:**

**Figure:**
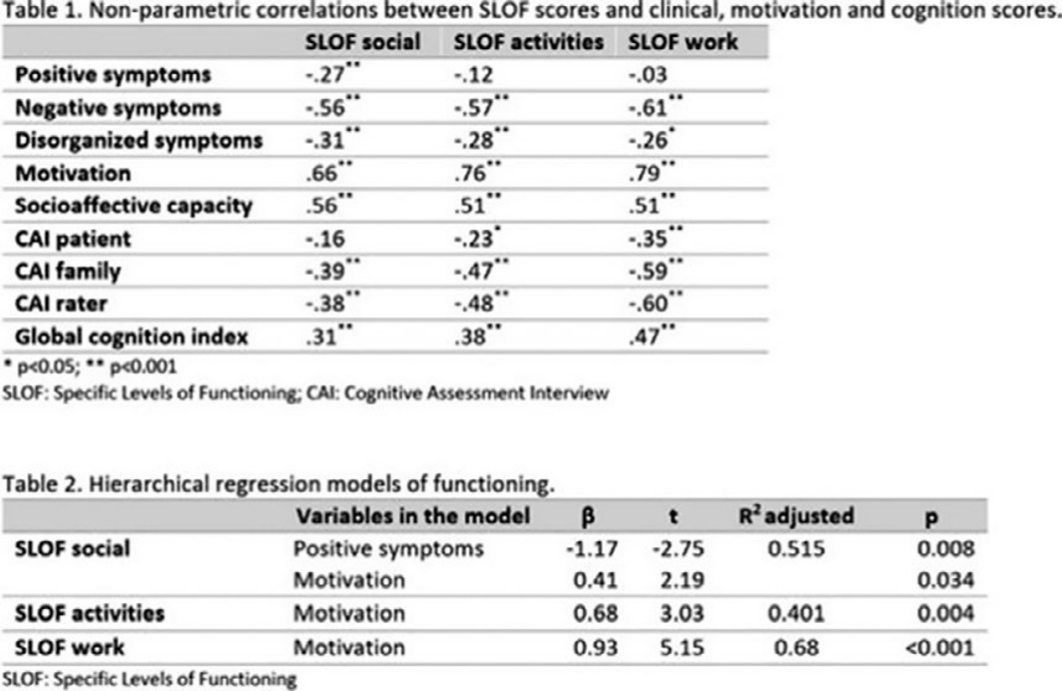

**Conclusions:**

Motivation has a great value as a predictor factor in social, activities and work functioning. Therefore, motivation should be considered as a target related to improving functioning in early intervention programmes for psychotic disorders.

**Disclosure of Interest:**

None Declared

